# Lifestyle Medicine Perspectives from Nursing in Community Care Setting: A Narrative Review

**DOI:** 10.3390/nursrep16040128

**Published:** 2026-04-10

**Authors:** Francesco Sacchini, Francesco Scerbo, Karolina Kowalcze, Paola Pantanetti, Sophia Russotto, Otilia Enache, Stefano Mancin, Cuc Thi Thu Nguyen, Diego Lopane, Francesca Marfella, Gabriele Caggianelli, Robert Krysiak, Fabio Petrelli, Giovanni Cangelosi

**Affiliations:** 1 Community Hospital of Sant’Elpidio a Mare, Local Health Authority AST Fermo, 63811 Sant’Elpidio a Mare, Italy; francescosacchini@libero.it; 2 Centre of Excellence for Nursing Scholarship, Order of Nurses of Rome, 00173 Rome, Italy; scerbofrancesco@gmail.com; 3 Department of Biomedicine and Prevention, University of Rome Tor Vergata, Via Cracovia n.50, 00133 Rome, Italy; 4 Department of Pediatrics in Bytom, Faculty of Health Sciences in Katowice, Medical University of Silesia, Stefana Batorego 15, 41-902 Bytom, Poland; kkowalcze@sum.edu.pl; 5 Department of Pathophysiology, Faculty of Medicine, Academy of Silesia, Rolna 43, 40-555 Katowice, Poland; 6 Local Health Authority AST Fermo, 63800 Fermo, Italy; paola.pantanetti@sanita.marche.it; 7 Department of Translation Medicine, University of Eastern Piedmont, 28100 Novara, Italy; 20015279@studenti.uniupo.it; 8 School of Pharmacy, Experimental Medicine and “Stefani Scuri” Public Health Department, University of Camerino, 62032 Camerino, Italy; otilia.enache94@gmail.com (O.E.); giovanni01.cangelosi@unicam.it (G.C.); 9 IRCCS Humanitas Research Hospital, 20089 Rozzano, Italy; stefano.mancin@humanitas.it (S.M.); diego.lopane@hunimed.eu (D.L.); 10 De Department of Pharmaceutical Administration and Economics, Hanoi University of Pharmacy, Hanoi 10000, Vietnam; cucnguyen.pharm@gmail.com; 11 Italian Coordination of Volunteer Nurses for Health Emergencies Association (CIVES), Via Agostino Depretis 70, 00184 Rome, Italy; direzioneoperativa@cives-odv.org; 12 Department of Healthcare Professions, Azienda Ospedaliera Complesso Ospedaliero San Giovanni Addolorata, 00184 Rome, Italy; caggianelligabriele@gmail.com; 13 Center of Excellence for Nursing Culture and Research, 00713 Rome, Italy; 14 Department of Internal Medicine and Clinical Pharmacology, Medical University of Silesia, Medyków 18, 40-752 Katowice, Poland

**Keywords:** family and community nurse, chronic care, lifestyle medicine, narrative review

## Abstract

**Background/Objectives**: Chronic diseases pose a major challenge for healthcare systems, requiring integrated, patient-centered approaches that combine clinical management, prevention, and self-care. Lifestyle Medicine (LM) and lifestyle in general offers complementary frameworks to address these needs. However, the potential integration of LM within community nursing—particularly through the role of Family and Community Nurse (FCN)—has not been comprehensively synthesized. This narrative review aimed to synthesize international evidence on the role of community nursing—particularly FCN—in integrating chronic care management and LM view. **Methods**: For quality assessment, a narrative review was conducted in accordance with the SANRA criteria to enable the integration of heterogeneous evidence and a comprehensive synthesis of this complex topic. Literature searches were performed in the PubMed–Medline database, and the final screening of references from included studies was used to identify relevant manuscripts. Primary studies published in English over the past ten years were screened and analyzed using the PICOS framework. Sixteen eligible studies were included in the final synthesis. **Results**: The included studies indicated that nurse-led community interventions in LM view were associated with improvements in self-management, treatment adherence, and selected clinical outcomes, such as blood pressure, glycated hemoglobin, and physical activity levels. Empowerment-based approaches and the use of digital or telehealth tools supported patient engagement and health literacy. At the organizational level, multidisciplinary collaboration, shared protocols, and professional leadership emerged as key factors in sustaining continuity and quality of care, while organizational fragmentation and limited training in behavioral counseling were commonly reported barriers. **Conclusions**: Community nursing, particularly through FCNs, plays a relevant role in integrating chronic care management and LM approaches, contributing to improved self-management, treatment adherence, and selected clinical outcomes. The evidence highlights the importance of empowerment-based interventions, digital support tools, and multidisciplinary collaboration in enhancing care continuity and patient engagement. Addressing organizational barriers and strengthening behavioral counseling training remain essential to support effective implementation in community settings.

## 1. Introduction

Chronic noncommunicable diseases (NCDs) are responsible for the majority of deaths worldwide, and their burden is rising due to aging populations, behavioral risk factors, and healthcare inequalities [1,2]. The concept of chronic care has gained central importance since the introduction of the Chronic Care Model (CCM) proposed by Wagner in the 1990s, which redefined the management of chronic conditions by integrating healthcare services, communities, and patients themselves [3]. In Italy, the application of this model has been analyzed by Petrelli et al., who highlighted its strengths and weaknesses across different regions [4]. This model emphasizes the need for a proactive, multidisciplinary, and patient-centered healthcare system that fosters patient self-management and ensures continuity of care [5]. From an epidemiological perspective, NCDs are a top public health priority, affecting about one in three adults in Economic Co-operation and Development (OECD) countries with major health and economic impacts [6]. Multimorbidity affects a substantial proportion of the population in Europe and Italy, particularly among older adults [7,8]. However, inadequate food knowledge among patients with chronic conditions still represents a major barrier to effective self-management and prevention [9]. The most common conditions include hypertension, osteoarthritis/arthritis, diabetes, and cardiovascular diseases [10]. The most recent National Chronicity Plan (Italian Ministry of Health) emphasizes the need for multidisciplinary, community-based care with a central role for nurses, whose profession has evolved from a predominantly technical function to a complex role encompassing clinical, educational, and coordination skills, as highlighted by the International Council of Nurses (ICNs) in promoting safe, evidence-based care and patient empowerment [11,12,13]. Key competencies include clinical management, leadership, working within multidisciplinary teams, and using digital technologies for monitoring and patient care [14]. European policy documents highlight the importance of nurses in strengthening chronicity-oriented health systems and developing advanced skills in community and primary care [15,16]. The Family and Community Nurse (FCN), introduced as a strategic resource, has been shown internationally to reduce hospitalizations, improve adherence, and promote healthy lifestyles [17,18]. Studies, including research from the University of Genoa, stress the need for a standardized FCN profile [19], and in Italy, Ministerial Decree 77/2022 institutionalized the FCN as a key professional in Community Health Centers, marking a shift to a structural role in community health innovation [20,21,22]. In recent years, Lifestyle Medicine (LM) and lifestyle behaviors more broadly have emerged as an innovative approach to the prevention and management of chronic diseases. According to the American College of Lifestyle Medicine (ACLM), LM applies evidence-based interventions such as healthy nutrition, regular physical activity, stress management, adequate sleep, avoidance of harmful substances, and promotion of positive social connections [23]. These interventions can reduce the risk of chronic conditions and improve outcomes in affected patients [24,25], with Italian research confirming that adherence to the Mediterranean Diet (MD) supports metabolic control and psychological well-being in type 2 diabetes (T2D) [26]. The World Health Organization (WHO) highlights that lifestyle changes could prevent 40% of cancers and 80% of cardiovascular diseases and diabetes [2]. Integrated LM programs globally reduce hospitalizations, improve quality of life, and lower healthcare costs [27,28]. Nurses are uniquely positioned to implement LM in practice, promoting healthy behaviors, supporting adherence, and developing personalized self-management programs [29], drawing on competencies in nutritional counseling, physical activity promotion, and digital monitoring tools [30]. In Italy, the FCN is ideally placed to bridge chronic care and LM, with specific responsibilities in health promotion and primary prevention [31]. International pilot experiences in nursing have shown that integrating LM interventions into care pathways reduces cardiovascular risk factors and improves patients’ physical and psychological well-being [32]. This narrative review addresses gaps in the literature on nurses’ roles, particularly the FCN, in chronic disease management and lifestyle interventions. Few studies have analyzed the intersection of nursing competencies, community-based care, and LM, highlighting the need for an integrated review to support practice and policy.

## 2. Materials and Methods

### 2.1. Study Design

This study adopts a narrative review design [33,34] and follows the Scale for the Evaluation of Narrative Review Articles (SANRA) to ensure quality accuracy, clarity, and scientific rigor (Checklist in Supplementary File S1). The objective of this work is to provide a comprehensive qualitative synthesis of the literature regarding the possible role of FCN in LM perspectives, with a focus on self-management, continuity of care, prevention, and territorial proximity in the management of chronic conditions.

### 2.2. Search Strategy and Research Questions

The research questions guiding this narrative review are the following:

- What is the role of LM in enhancing the effectiveness of chronic care pathways delivered in community settings?
- How could community-based nursing interventions could contribute to improving clinical outcomes and self-management in patients with chronic diseases?
- Which models of community care and nursing-led interventions demonstrate measurable benefits in adherence, patient empowerment, and reduction in hospital-centered care?

#### Patient Intervention Comparison Outcome Study (PICOS)

The review was structured according to the PICOS framework [35], as outlined below:

Population (P): adults with chronic diseases in community settings, including individuals with cardiovascular, metabolic, or behavioral risk conditions, as well as healthcare professionals and organizations involved in the care process;

Intervention (I): community nursing strategies from an LM perspective, defined based on concrete operational criteria:

- Inclusion of at least one of the main LM pillars: nutrition, physical activity, stress management, sleep, and avoidance of harmful substances;
- Focus on prevention, promotion of self-care, and empowerment of participants;
- Clearly defined delivery modalities: individual, group, digital, or community-based interventions.

Comparison (C): standard care, absence of intervention, or unstructured support programs not based on the principles of LM;

Outcome (O): improvement in self-management for both patients and professionals, adherence to recommendations, quality of life, clinical stability, or reduction in preventable hospitalizations. Outcomes include clinical measures (metabolic parameters, blood pressure, weight, blood glucose), behavioral measures (adherence to diet, physical activity, stress management), and psychosocial measures (empowerment, self-care, quality of life);

Study type (S): primary studies, including experimental, observational, and qualitative/quantitative designs.

### 2.3. Inclusion and Screening Criteria

The eligibility criteria included primary studies published in English over the last ten years and focused on chronic care, community nursing, or lifestyle-based strategies applied to chronic conditions, according to the PICOS framework and the research questions developed. Exclusion criteria ruled out editorials, commentaries, conference abstracts, reviews, and protocol papers. The literature search was performed on 7 January 2026, using the PubMed database via Medline, applying Boolean operators to identify studies relevant to the research question and the specific search strategy (Supplementary File S2). The screening process was conducted independently by two reviewers; disagreements were resolved through collegial discussion. Final screening of the references of the included studies was conducted for the retrieved relevant manuscripts.

### 2.4. Data Synthesis and Risk of Bias

All included studies were first charted by author, year, setting, population, intervention, outcomes, and key findings, enabling a structured synthesis. A narrative approach was then applied to integrate emerging concepts and highlight convergences and differences across the selected studies. Methodological quality and risk of bias were independently assessed by two reviewers using the Critical Appraisal Skills Program (CASP) checklist [36], applied according to the study design (experimental, observational, or qualitative), as detailed in Supplementary File S3. This quality assessment informed the narrative synthesis, highlighting which findings are supported by robust evidence and which should be interpreted with caution due to methodological limitations, ensuring transparency and reliability in the overall analysis.

## 3. Results

**Figure 1:**
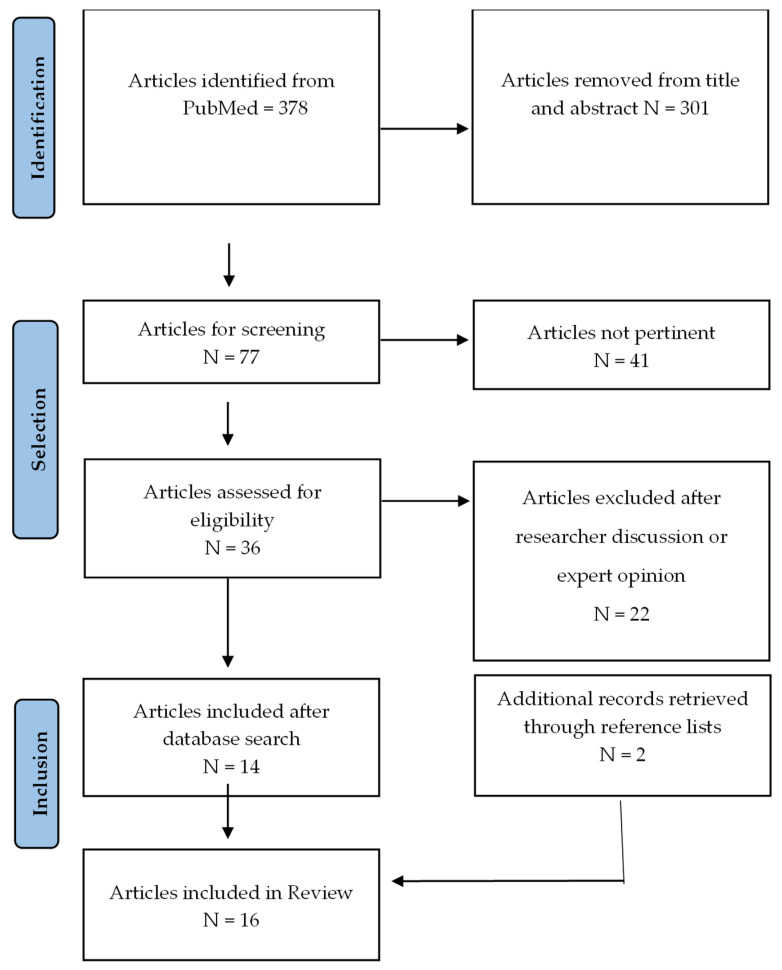
Flowchart Selection.

Using the search strategy, 378 records were identified in the PubMed database. After title and abstract screening, 301 studies were excluded as they did not meet the research topic or eligibility criteria. Seventy-seven full-text articles were subsequently assessed for eligibility. Of these, 41 were excluded as not pertinent, and a further 22 full-text articles were excluded after collegial discussion and expert consultation. As a result, 14 studies were included from the database search. In addition, screening the reference lists of the included studies identified two other studies. Overall, 16 primary studies were included in this narrative review (Figure 1). All included studies received a positive rating according to the CASP checklist’s minimum criteria [36].

### 3.1. Studies Selection

Among the sixteen primary studies included [37,38,39,40,41,42,43,44,45,46,47,48,49,50,51,52], the United States was the most represented setting with 6 studies [39,40,42,47,48,50], followed by Australia (*n* = 2; [43,51]). The remaining studies were conducted in China [38], the Netherlands [49], Saudi Arabia [45], Portugal [37], Finland [46], the United Kingdom [44], Iran [52], and Canada [41] (one study each). Overall, the studies reflect a broad international distribution across North America, Europe, Oceania, Asia, and the Middle East, highlighting the global relevance of community-based chronic care and lifestyle-oriented interventions. With regard to study design, ten studies were experimental, quasi-experimental, or used an implementation framework [37,38,40,41,42,43,47,50,51,52]; six were observational [39,44,45,46,48,49]. This methodological heterogeneity provides a comprehensive perspective on both the effectiveness and feasibility of community nursing and lifestyle medicine interventions across different healthcare settings. The main characteristics of the included studies are summarized in Table 1 and Table 2.

### 3.2. Patient-Level Interventions: Lifestyle Change, Empowerment and Self-Management Support

A core group of studies focused on patient-level strategies to support lifestyle modification, empowerment, and day-to-day self-management in chronic conditions. Several randomized or quasi-experimental interventions reported that structured education, behavioral coaching, and personalized follow-up were linked to improvements in self-efficacy, adherence, and certain clinical parameters over time, particularly in diabetes and hypertension [38,39,49,50]. Digital and telehealth-supported approaches were described as facilitating engagement and self-monitoring behaviors, enhancing risk-factor awareness, and supporting daily routines [42]. Nurse-led programs, in particular, were associated with changes in adherence, self-management skills, and short-term improvements in medication knowledge, confidence, and behavior [37,41,46,49]. Although the magnitude and duration of observed effects varied across settings, the studies consistently emphasized that patient activation and empowerment support lifestyle-oriented chronic care. Overall, the evidence indicates that personalized education, support from family or community resources, and continuity of contact—whether in person or digitally mediated—may play an important role in promoting lifestyle outcomes and self-management within community-based care models.

### 3.3. System-Level and Organizational Interventions

A second group of studies examined system-level strategies intended to reorganize services, integrate community resources, and strengthen multidisciplinary care for chronic conditions. Implementation studies and organizational redesign projects indicated that structured care models, such as coordinated outreach programs, multidisciplinary pathways, and team-based chronic care models, were linked to improvements in continuity, preventive activities, and workflow efficiency in primary care [43,47,51,52]. When combined with facilitation, leadership engagement, and shared protocols, these approaches appeared to support more coherent delivery of self-management support and the role of nurses within interdisciplinary teams [40,48]. At the same time, system-level interventions highlighted the influence of organizational culture, communication, and roles clarity in sustaining innovations and embedding self-management support into routine practice [44]. The literature also pointed out persistent structural barriers, including time constraints, fragmented pathways, and limited funding mechanisms, which may limit scalability and long-term sustainability [43,48,51]. Collectively, these findings suggest that system-level strategies can complement patient-level interventions, particularly when supported by governance, resource allocation, and a shared vision of community-based chronic care.

### 3.4. Barriers, Professional Perspectives, and Challenges

A final group of studies addressed contextual and psychosocial factors affecting self-management and lifestyle change, identifying barriers at patient, provider, and community levels. Qualitative and cross-sectional studies highlighted that lifestyle modification can be limited by motivational challenges, self-regulation skills, emotional burden, socioeconomic constraints, and environmental factors such as climate and resource accessibility [44,45]. Professional perspectives indicated that time pressures, fragmented care pathways, and limited training in behavior change techniques may reduce the consistency and depth of self-management support in routine practice [44,48]. Patient activation and perceived health status emerged as relevant determinants of engagement, with higher activation generally associated with healthier behaviors and more favorable self-management trajectories [41]. These studies collectively indicate that, even with structured interventions or organizational redesign, the outcomes of chronic care models depend on relational continuity, patient empowerment, and the availability of community resources. The findings illustrate the interplay between patient-level strategies, organizational structures, and contextual factors, providing a descriptive foundation for interpreting the broader implications without introducing evaluative statements, as discussed in the following section.

## 4. Discussion

Community nursing, including the FNC, emerges as a strategic lever for integrating chronic care management and LM, promoting patient self-management, empowerment, and healthy behaviors, while contributing to improved clinical outcomes and more efficient, coordinated service delivery. Randomized and quasi-experimental studies reported improvements in treatment adherence, disease knowledge, and self-management capacity, with downstream effects on clinical indicators such as glycated hemoglobin and blood pressure [38,39,49,50]. From a governance perspective, these results support international evidence indicating that self-care facilitation is a high-value nursing function capable of enhancing system performance through patient empowerment and behavioral change [53]. Through relational proximity and continuity of care, FNC operationalizes the principles of chronic care at the community level by integrating educational and motivational strategies—such as motivational interviewing, personalized care planning, and structured follow-up—into routine practice [54]. Across the sixteen studies analyzed, lifestyle-oriented nursing interventions—including nutritional counseling, physical activity promotion, telemonitoring, and digital support—were associated with measurable improvements in self-efficacy and quality of life [55]. These findings highlight LM not merely as a clinical approach but as a system-level strategy for preventing disease progression and reducing long-term care burden through behavioral modification and self-care development. Digital health and telecare platforms emerged as enabling infrastructures for service integration and continuity, improving patient engagement, reducing geographical barriers, and supporting coordination between hospital and community settings [39,42]. From a managerial standpoint, digital nursing interventions enhance scalability and sustainability by extending the reach of care without proportionally increasing resource consumption, while reinforcing patient activation and health literacy across clinical and social domains [56,57]. However, their effectiveness depends on alignment with organizational structures and care pathways. Implementation studies demonstrated that reorganizing community services according to integrated CCM—supported by nursing leadership, shared protocols, and interprofessional collaboration—enhances continuity, accountability, and overall quality of care [43,47,52]. These findings are consistent with broader international evidence emphasizing that coordinated, interdisciplinary models are essential for managing chronicity in resource-constrained health systems [58,59]. Despite these advantages, the review also highlights persistent structural and organizational barriers. Fragmentation of services, limited time allocation for therapeutic education, insufficient training in behavioral change techniques, and the lack of outcome indicators capturing empowerment and quality of life remain critical obstacles to full implementation [60,61]. Qualitative studies further emphasize that relational continuity and therapeutic alliance—grounded in trust, communication, and motivational support—are not merely clinical attributes but organizational conditions that require appropriate staffing models, workload distribution, and institutional recognition [62,63]. Another recurrent theme concerns the family and community dimension of chronic care pathways. Interventions actively engaging family members and informal support networks demonstrate greater sustainability in maintaining healthy behaviors over time, reinforcing the role of community-based services as mediators between health systems and social environments. From a policy perspective, this highlights the importance of designing care models that extend beyond the individual patient to encompass social and environmental contexts. Overall, the evidence positions the FNC as a pivotal professional figure capable of aligning the CCM’s organizational principles—such as coordination, proactivity, and continuity—with the LM’s preventive and behavioral focus. This integration supports a paradigm shift from disease-centered care to health-oriented systems, in which nurses function as facilitators of change across the entire care continuum. Looking forward, the implications for health policy and service management are substantial. Strengthening community nursing requires targeted investment in advanced education, leadership development, and organizational redesign to ensure protected time, dedicated spaces, and appropriate tools for therapeutic education and behavioral support [64,65]. The findings of this narrative review therefore demonstrate that community nursing is not only a clinical resource but a strategic asset for health system sustainability. Embedding these competencies within community services—supported by coherent policy frameworks and continuous professional development—is essential to deliver chronic care that is effective, equitable, and resilient in the long term.

### 4.1. Future Research Perspectives

Future research should prioritize large-scale, multicenter, and longitudinal studies capable of informing health system decision-making by assessing both long-term clinical and psychosocial outcomes—such as empowerment, self-efficacy, and quality of life—using both qualitative and quantitative outcome measures to facilitate cross-study comparisons and synthesis [66,67]. Although current evidence suggests that empowerment-based approaches can significantly enhance patients’ perceived control and health behaviors, heterogeneity in study designs continues to limit generalizability and transferability to policy and practice [68]. Further investigations are needed to clarify the role of health literacy as a mediating factor in self-management and lifestyle adherence, particularly in community settings where nurses act as primary educators and facilitators of change [69,70]. These findings underscore the importance of developing evidence-based strategies to strengthen FNC’s educational, communicative, and relational competencies, aligning workforce development with evolving population needs. In parallel, future research should deepen the analysis of nursing processes within chronic care frameworks, identifying which organizational, behavioral, and contextual elements are most effective in sustaining long-term outcomes and patient engagement [71,72]. Comparative implementation studies across different countries and health systems are also essential to evaluate the contextual facilitators and barriers to integrated, people-centered chronic care and to identify shared values, governance mechanisms, and organizational models that promote equity, sustainability, and scalability [73,74]. Overall, future investigations should integrate clinical, behavioral, and organizational dimensions, adopting mixed-method designs and standardized outcome measures to generate robust evidence on the long-term impact of nurse-led chronic care pathways and the integration of LM perspectives within community health systems.

### 4.2. Strengths and Limitations

The integration of community nursing within chronic care in LM view has generated valuable insights into the evolving role of nurses in promoting self-management and health behavior change. This review synthesizes recent international evidence—spanning experimental, observational, and qualitative studies—to highlight both clinical outcomes, such as improvements in HbA1c, blood pressure, and treatment adherence, and psychosocial dimensions, including greater self-efficacy and patient empowerment. It also emphasizes organizational factors, such as nursing leadership, shared protocols, and interprofessional collaboration, that strengthen care continuity and have implications for practice and policy. The main limitations of the study are the use of a single database for data extraction and the language restriction, with selection limited to English. In addition, the choice of a narrative review approach was driven by the marked heterogeneity of the included studies, which differ substantially in terms of care settings, target populations, types of intervention, and outcome measures. Such variability makes the application of the more stringent criteria typical of a systematic review challenging and limits the feasibility of a quantitative comparative synthesis. As a narrative review, the absence of a quantitative meta-analysis prevents the comparative estimation of intervention effects. It should be emphasized that, although all studies received a positive rating, this rating reflects the minimum quality criteria applied and the study selection process, and does not preclude the presence of potential methodological limitations in the individual studies. Moreover, the heterogeneity of study settings, populations, and outcomes reduces the generalizability of findings. In this regard, it is important to note that no studies from Africa, South America, the Caribbean, or Australia were identified, resulting in a geographical imbalance and underrepresentation of several world regions. This may further limit the applicability of the findings, as contextual, cultural, and healthcare system differences across regions could influence the observed outcomes. Additionally, possible language restrictions and publication bias may have excluded relevant studies. Furthermore, many investigations feature short follow-up periods and employ non-standardized measures of empowerment or quality of life. These limitations highlight the need for future systematic reviews and longitudinal studies with preregistered protocols, harmonized core outcome sets, and longer observation periods, to confirm the robustness of current evidence and identify key modulators such as intervention intensity, active components, and delivery modalities.

## 5. Conclusions

Community nursing, particularly FNC, could play a pivotal role in integrating chronic care management with lifestyle medicine. Nurse-led interventions could enhance patient empowerment, self-management, and clinical outcomes, while supporting behavioral change through education and continuity of care, and specific digital tools could further strengthen service integration in multi-dimensional/multi-disciplinary perspectives. Addressing barriers such as fragmented pathways and training gaps could require workforce development and supportive policy frameworks. Systematic integration of community nursing could make chronic care more effective, equitable, and sustainable, promoting a preventive, person-centered model for resilient health systems.

## Figures and Tables

**Table 1:** Characteristics of the Included Studies.

Characteristic	Frequency (*n* = 16)	Percentage
Publication year		
2024	1	6.3%
2022	1	6.3%
2021	2	12.5%
2020	5	31.3%
2019	2	12.5%
2018	1	6.3%
2017	4	25%
Country		
USA	6	37.5%
Australia	2	12.5%
China	1	6.3%
Netherlands	1	6.3%
Saudi Arabia	1	6.3%
Portugal	1	6.3%
Finland	1	6.3%
United Kingdom	1	6.3%
Iran	1	6.3%
Canada	1	6.3%
Study Design		
Experimental/Quasi-experimental	4	25%
Observational	3	18.8%
Qualitative	5	31.3%
Implementation/Quality Improvement	4	25%
Quality of study		
Positive	16	100%
Negative	0	0%
Unknows	0	0%

**Table 2:** Summary of Included Studies.

First Author and Year	Country	Study Design	Sample	Intervention	Objective	Results	Limitations
Oliveira et al. [37], 2024	Portugal	Quality improvement	8 nurses, 140 patients	Nurse-led adherence program	Improve medication adherence using EBP audits	Significant improvement in adherence practices	Single setting; short follow-up
Yang et al. [38], 2022	China	RCT	136 older adults	Nurse-led medication self-management	Improve adherence and self-efficacy	Improved short-term adherence and knowledge	Short follow-up; self-report bias
Higa et al. [39], 2021	USA	Observational	7 patients and caregivers	Telehealth and family support (MOD-P)	Improve diabetes self-management	Improved HbA1c and DSM engagement	Small sample; no control group
Casanova et al. [40], 2021	USA	Multi-case implementation study	10 organizations	Obesity care model	Implement system-level CCM	Improved coordination and workflows	No control; resource intensive
Hersson-Edery et al. [41], 2021	Canada	Qualitative participatory descriptive study	14 patients and staff	Empowerment group program (DEGP)	Describe feasibility and components	Feasible, supported empowerment	Single site; no clinical outcomes
Nagykaldi et al. [42], 2020	USA	Quasi-experimental implementation Study	3 rural counties	Community outreach model	Improve preventive care delivery	+20% preventive services delivered	No control group; rural only
Redfern et al. [43], 2020	Australia	RCT	934 patients	Digital lifestyle tool	Improve adherence and risk factors	Improved PA and e-literacy; limited clinical effect	Underpowered; mixed adoption
Harris et al. [44], 2020	UK	Qualitative descriptive study	21 professionals	SMS practices exploration	Examine SMS approaches	SMS varied, relationship-dependent	Limited generalizability
Alshammari et al. [45], 2020	Saudi Arabia	Cross-sectional descriptive study	250 patients	Barriers to lifestyle change	Identify barriers	Low PA; environmental and motivational barriers	Self-report bias; single city
Tusa et al. [46], 2020	Finland	Cross-sectional analytical study	597 patients	PAM assessment	Analyze activation and SRH	Higher PAM linked to better SRH	Cross-sectional; self-report
Dickinson et al. [47], 2019	USA	Cluster randomized implementation trial	36 practices	SMS + tech + facilitation	Improve SMS and HbA1c	Improved SMS and HbA1c (per-protocol)	Variability in adoption
Jortberg et al. [48], 2019	USA	Cross-sectional mixed-methods study	36 practices	SMS implementation	Identify determinants of SMS	Culture, PCMH and care managers key	Observational; baseline only
Westland et al. [49], 2018	Netherlands	Qualitative observational	78 consultations	Routine nurse SMS	Analyze SMS content	SMS inconsistent and biomedical-focused	Limited representativeness
Gotwals [50], 2018	USA	Quasi-experimental controlled study	92 nurses	Nutrition education	Improve counseling self-efficacy	Improved self-efficacy scores	No patient outcomes
Volker et al. [51], 2017	Australia	Qualitative implementation study	40 stakeholders	CVD prevention model	Assess implementation	Teamwork and system barriers identified	Regional sample only
Daniali et al. [52], 2017	Iran	RCT	146 women	SMS education	Improve BP and behaviors	Improved PA, self-efficacy and BP	Women only; short follow-up

Legend. SMS: Self-Management Support; DSM: Diabetes Self-Management; PA: Physical Activity; SRH: Self-Rated Health; PAM: Patient Activation Measure; RCT: Randomized Controlled Trial; CCM: Chronic Care Model; EBP: Evidence-Based Practice; PCMH: Patient-Centered Medical Home; HbA1c: glycated hemoglobin; BP: Blood Pressure; MOD-P: Modified Program.

## Data Availability

The data supporting this research are available upon request from the corresponding author for data protection reasons.

## Supplementary Materials

The following supporting information can be downloaded at: https://www.mdpi.com/article/10.3390/nursrep16040128/s1, File S1: SANRA Checklist; File S2: Search Strategy; File S3: CASP checklist.

## Public Involvement Statement

No public involvement in any aspect of this research.

## Guidelines and Standards Statement

This study follows the Scale for the Evaluation of Narrative Review Articles (SANRA) [33] for transparency and reproducibility.
