# Supplementary material for: Lifestyle Medicine Perspectives from Nursing in Community Care Setting: A Narrative Review
Source: Nurs Rep. 2026 Apr 10;16(4):128. doi: 10.3390/nursrep16040128 (PMC13118283; doi:10.3390/nursrep16040128)
Supplement: Supplementary file 1 [file nursrep-16-00128-s001.zip › nursrep-4176729-supplementary.pdf]

# SUPPLEMENTARY FILES

**S1:** SANRA Checklist.

## Scale for the Assessment of Narrative Review Articles – SANRA

Please rate the quality of the narrative review article in question, using categories 0–2 on the following scale. For each aspect of quality, please choose the option which best fits your evaluation, using categories 0 and 2 freely to imply general low and high quality. These are not intended to imply the worst or best imaginable quality.

### 1) Justification of the article's importance for the readership

The importance is not justified. — 0
The importance is alluded to, but not explicitly justified. — 1
The importance is explicitly justified. — 2

☐

### 2) Statement of concrete aims or formulation of questions

No aims or questions are formulated. — 0
Aims are formulated generally but not concretely or in terms of clear questions. — 1
One or more concrete aims or questions are formulated. — 2

☐

### 3) Description of the literature search

The search strategy is not presented. — 0
The literature search is described briefly. — 1
The literature search is described in detail, including search terms and inclusion criteria. — 2

☐

### 4) Referencing

Key statements are not supported by references. — 0
The referencing of key statements is inconsistent. — 1
Key statements are supported by references. — 2

☐

### 5) Scientific reasoning

*(e.g., incorporation of appropriate evidence, such as RCTs in clinical medicine)*

The article's point is not based on appropriate arguments. — 0
Appropriate evidence is introduced selectively. — 1
Appropriate evidence is generally present. — 2

☐

### 6) Appropriate presentation of data

*(e.g., absolute vs relative risk; effect sizes without confidence intervals)*

Data are presented inadequately. — 0
Data are often not presented in the most appropriate way. — 1
Relevant outcome data are generally presented appropriately. — 2

☐

**Sumscore** ☐

**Fig. 1** SANRA - Scale

Score: 1: 2; 2:2; 3:2; 4:2; 5:2; 6:2.

SumScore: 12.

**S2**: Search strategy update January 07, 2026 to Pubmed from Medline.

( "chronic disease"[tiab] OR "disease management"[tiab] OR "integrated care"[tiab] OR "integrated health care"[tiab] OR "continuity of care"[tiab] OR "patient care management"[tiab] OR "care coordination"[tiab] OR "patient-centered care"[tiab] OR "population health"[tiab] ) AND ( "community health nursing"[tiab] OR "primary health care"[tiab] OR "family nursing"[tiab] OR "public health nursing"[tiab] OR "community health services"[tiab] OR "home care"[tiab] OR "community-based nursing"[tiab] OR "community nursing"[tiab] OR "nurse's role"[tiab] ) AND ( "life style" [tiab] OR "lifestyle"[tiab] OR "health promotion"[tiab] OR "preventive health"[tiab] OR "health behavior"[tiab] OR "self-care" [tiab] OR "patient education"[tiab] OR "health knowledge"[tiab] OR "holistic health"[tiab] OR "wellness"[tiab] OR "lifestyle medicine" [tiab] OR "behavioral medicine"[tiab] ) Sort by: Most Recent

*Filters*: last 10 years

Total results: 378

## **S3:** CASP Checklist.

Randomised Controlled Trials (RCTs)

| Reference: Yang et al. / 2022 | | | | |
|---|---|---|---|---|
| Section A Is the basic study design valid for a randomised controlled trial? | | | | |
| N | Item | Yes | Can't Tell | No |
| 1 | Did the study address a clearly formulated research question? | X | | |
| 2 | Was the assignment of participants to interventions randomised? | X | | |
| 3 | Were all participants who entered the study accounted for at its conclusion? | X | | |
| Section B Was the study methodologically sound? | | | | |
| N | Item | Yes | Can't Tell | No |
| 4a | Were the participants 'blind' to intervention they were given? | X | | |
| 4b | Were the investigators 'blind' to the intervention they were giving to participants? | X | | |
| 4c | Were the people assessing/analysing outcome/s 'blinded'? | X | | |
| 5 | Were the study groups similar at the start of the randomised controlled trial? | X | | |
| 6 | Apart from the experimental intervention, did each study group receive the same level of care (that is, were they treated equally)? | X | | |
| Section C: What are the results? | | | | |
| N | Item | Yes | Can't Tell | No |
| 7 | Were the effects of intervention reported comprehensively? | X | | |

| | | | | |
|---|---|---|---|---|
| 8 | Was the precision of the estimate of the intervention or treatment effect reported? | X | | |
| 9 | Do the benefits of the experimental intervention outweigh the harms and costs? | X | | |
| Section D: Will the results help locally? | | | | |
| N | Item | Yes | Can't Tell | No |
| 10 | Can the results be applied to your local population/in your context? | X | | |
| 11 | Would the experimental intervention provide greater value to the people in your care than any of the existing interventions? | X | | |
| APPRAISAL SUMMARY | | | | |
| Positive/Methodologically sound | Negative/Relatively poor methodology | Unknowns | | |
| X | | | | |

| | | | | |
|---|---|---|---|---|
| Reference: Redfern et al. / 2020 | | | | |
| Section A Is the basic study design valid for a randomised controlled trial? | | | | |
| N | Item | Yes | Can't Tell | No |
| 1 | Did the study address a clearly formulated research question? | X | | |
| 2 | Was the assignment of participants to interventions randomised? | X | | |
| 3 | Were all participants who entered the study accounted for at its conclusion? | X | | |
| Section B Was the study methodologically sound? | | | | |
| N | Item | Yes | Can't Tell | No |
| 4a | Were the participants 'blind' to intervention they were given? | X | | |
| 4b | Were the investigators 'blind' to the intervention they were giving to participants? | X | | |
| 4c | Were the people assessing/analysing outcome/s 'blinded'? | X | | |
| 5 | Were the study groups similar at the start of the randomised controlled trial? | X | | |
| 6 | Apart from the experimental intervention, did each study group receive the same level of care (that is, were they treated equally)? | X | | |
| Section C: What are the results? | | | | |
| N | Item | Yes | Can't Tell | No |
| 7 | Were the effects of intervention reported comprehensively? | X | | |
| 8 | Was the precision of the estimate of the intervention or treatment effect reported? | X | | |
| 9 | Do the benefits of the experimental intervention outweigh the harms and costs? | X | | |

| Section D: Will the results help locally? | | | | |
|---|---|---|---|---|
| N | Item | Yes | Can't Tell | No |
| 10 | Can the results be applied to your local population/in your context? | X | | |
| 11 | Would the experimental intervention provide greater value to the people in your care than any of the existing interventions? | X | | |
| APPRAISAL SUMMARY | | | | |
| Positive/Methodologically sound | Negative/Relatively poor methodology | Unknowns | | |
| X | | | | |

| Reference: Daniali et al. / 2017 | | | | |
|---|---|---|---|---|
| Section A Is the basic study design valid for a randomised controlled trial? | | | | |
| N | Item | Yes | Can't Tell | No |
| 1 | Did the study address a clearly formulated research question? | X | | |
| 2 | Was the assignment of participants to interventions randomised? | X | | |
| 3 | Were all participants who entered the study accounted for at its conclusion? | X | | |
| Section B Was the study methodologically sound? | | | | |
| N | Item | Yes | Can't Tell | No |
| 4a | Were the participants 'blind' to intervention they were given? | | X | |
| 4b | Were the investigators 'blind' to the intervention they were giving to participants? | | X | |
| 4c | Were the people assessing/analysing outcome/s 'blinded'? | | X | |
| 5 | Were the study groups similar at the start of the randomised controlled trial? | X | | |
| 6 | Apart from the experimental intervention, did each study group receive the same level of care (that is, were they treated equally)? | X | | |
| Section C: What are the results? | | | | |
| N | Item | Yes | Can't Tell | No |
| 7 | Were the effects of intervention reported comprehensively? | X | | |
| 8 | Was the precision of the estimate of the intervention or treatment effect reported? | X | | |
| 9 | Do the benefits of the experimental intervention outweigh the harms and costs? | X | | |
| Section D: Will the results help locally? | | | | |
| N | Item | Yes | Can't Tell | No |

| | | | | |
|---|---|---|---|---|
| 10 | Can the results be applied to your local population/in your context? | X | | |
| 11 | Would the experimental intervention provide greater value to the people in your care than any of the existing interventions? | X | | |
| APPRAISAL SUMMARY | | | | |
| Positive/Methodologically sound | Negative/Relatively poor methodology | Unknowns | | |
| X | | | | |

| | | | | |
|---|---|---|---|---|
| Reference: Gotwals et al. / 2018 | | | | |
| Section A Is the basic study design valid for a randomised controlled trial? | | | | |
| N | Item | Yes | Can't Tell | No |
| 1 | Did the study address a clearly formulated research question? | X | | |
| 2 | Was the assignment of participants to interventions randomised? | | X | |
| 3 | Were all participants who entered the study accounted for at its conclusion? | X | | |
| Section B Was the study methodologically sound? | | | | |
| N | Item | Yes | Can't Tell | No |
| 4a | Were the participants 'blind' to intervention they were given? | | | X |
| 4b | Were the investigators 'blind' to the intervention they were giving to participants? | | X | |
| 4c | Were the people assessing/analysing outcome/s 'blinded'? | | X | |
| 5 | Were the study groups similar at the start of the randomised controlled trial? | X | | |
| 6 | Apart from the experimental intervention, did each study group receive the same level of care (that is, were they treated equally)? | X | | |
| Section C: What are the results? | | | | |
| N | Item | Yes | Can't Tell | No |
| 7 | Were the effects of intervention reported comprehensively? | X | | |
| 8 | Was the precision of the estimate of the intervention or treatment effect reported? | X | | |
| 9 | Do the benefits of the experimental intervention outweigh the harms and costs? | X | | |
| Section D: Will the results help locally? | | | | |
| N | Item | Yes | Can't Tell | No |
| 10 | Can the results be applied to your local population/in your context? | X | | |

| | | | | |
|---|---|---|---|---|
| 11 | Would the experimental intervention provide greater value to the people in your care than any of the existing interventions? | X | | |
| APPRAISAL SUMMARY | | | | |
| Positive/Methodologically sound | Negative/Relatively poor methodology | Unknowns | | |
| X | | | | |

Qualitative Studies

| | | | | |
|---|---|---|---|---|
| Reference: Westland et al. / 2021 | | | | |
| Section A: Are the result valid? | | | | |
| N | Item | Yes | Can't Tell | No |
| 1 | Was there a clear statement of the aims of the research? | X | | |
| 2 | Is a qualitative methodology appropriate? | X | | |
| 3 | Was the research design appropriate to address the aims of the research? | X | | |
| 4 | Was the recruitment strategy appropriate to the aims of the research? | X | | |
| 5 | Was the data collected in a way that addressed the research issue? | X | | |
| 6 | Has the relationship between researcher and participants been adequately considered? | | X | |
| Section B: What are the results? | | | | |
| N | Item | Yes | Can't Tell | No |
| 7 | Have ethical issues been taken into consideration? | X | | |
| 8 | Was the data analysis sufficiently rigorous? | X | | |
| 9 | Is there a clear statement of findings? | X | | |
| Section C: Will the results help locally? | | | | |
| N | Item | Yes | Can't Tell | No |
| 10 | How valuable is the research? | X | | |
| APPRAISAL SUMMARY | | | | |
| Positive/Methodologically sound | Negative/Relatively poor methodology | | | Unknowns |
| X | | | | |

| | | | | |
|---|---|---|---|---|
| Reference: Hersson-Edery et al. / 2021 | | | | |
| Section A: Are the result valid? | | | | |
| N | Item | Yes | Can't Tell | No |
| 1 | Was there a clear statement of the aims of the research? | X | | |

| | | | | |
|---|---|---|---|---|
| 2 | Is a qualitative methodology appropriate? | X | | |
| 3 | Was the research design appropriate to address the aims of the research? | X | | |
| 4 | Was the recruitment strategy appropriate to the aims of the research? | X | | |
| 5 | Was the data collected in a way that addressed the research issue? | X | | |
| 6 | Has the relationship between researcher and participants been adequately considered? | X | | |
| Section B: What are the results? | | | | |
| N | Item | Yes | Can't Tell | No |
| 7 | Have ethical issues been taken into consideration? | X | | |
| 8 | Was the data analysis sufficiently rigorous? | X | | |
| 9 | Is there a clear statement of findings? | X | | |
| Section C: Will the results help locally? | | | | |
| N | Item | Yes | Can't Tell | No |
| 10 | How valuable is the research? | X | | |
| APPRAISAL SUMMARY | | | | |
| Positive/Methodologically sound | Negative/Relatively poor methodology | | | Unknowns |
| X | | | | |

| Reference: Harris et al. / 2020 | | | | |
|---|---|---|---|---|
| Section A: Are the result valid? | | | | |
| N | Item | Yes | Can't Tell | No |
| 1 | Was there a clear statement of the aims of the research? | X | | |
| 2 | Is a qualitative methodology appropriate? | X | | |
| 3 | Was the research design appropriate to address the aims of the research? | X | | |
| 4 | Was the recruitment strategy appropriate to the aims of the research? | X | | |
| 5 | Was the data collected in a way that addressed the research issue? | X | | |
| 6 | Has the relationship between researcher and participants been adequately considered? | X | | |
| Section B: What are the results? | | | | |
| N | Item | Yes | Can't Tell | No |
| 7 | Have ethical issues been taken into consideration? | X | | |
| 8 | Was the data analysis sufficiently rigorous? | X | | |
| 9 | Is there a clear statement of findings? | X | | |
| Section C: Will the results help locally? | | | | |

| N | Item | Yes | Can't Tell | No |
|---|---|---|---|---|
| 10 | How valuable is the research? | X | | |
| APPRAISAL SUMMARY | | | | |
| Positive/Methodologically sound | Negative/Relatively poor methodology | | | Unknowns |
| X | | | | |

| Reference: Westland et al. / 2018 | | | | |
|---|---|---|---|---|
| Section A: Are the result valid? | | | | |
| N | Item | Yes | Can't Tell | No |
| 1 | Was there a clear statement of the aims of the research? | X | | |
| 2 | Is a qualitative methodology appropriate? | X | | |
| 3 | Was the research design appropriate to address the aims of the research? | X | | |
| 4 | Was the recruitment strategy appropriate to the aims of the research? | X | | |
| 5 | Was the data collected in a way that addressed the research issue? | X | | |
| 6 | Has the relationship between researcher and participants been adequately considered? | | X | |
| Section B: What are the results? | | | | |
| N | Item | Yes | Can't Tell | No |
| 7 | Have ethical issues been taken into consideration? | X | | |
| 8 | Was the data analysis sufficiently rigorous? | X | | |
| 9 | Is there a clear statement of findings? | X | | |
| Section C: Will the results help locally? | | | | |
| N | Item | Yes | Can't Tell | No |
| 10 | How valuable is the research? | X | | |
| APPRAISAL SUMMARY | | | | |
| Positive/Methodologically sound | Negative/Relatively poor methodology | | | Unknowns |
| X | | | | |

| Reference: Volker et al. / 2017 | | | | |
|---|---|---|---|---|
| Section A: Are the result valid? | | | | |
| N | Item | Yes | Can't Tell | No |
| 1 | Was there a clear statement of the aims of the research? | X | | |
| 2 | Is a qualitative methodology appropriate? | X | | |
| 3 | Was the research design appropriate to address the aims of the research? | X | | |

| | | | | |
|---|---|---|---|---|
| 4 | Was the recruitment strategy appropriate to the aims of the research? | X | | |
| 5 | Was the data collected in a way that addressed the research issue? | X | | |
| 6 | Has the relationship between researcher and participants been adequately considered? | X | | |
| Section B: What are the results? | | | | |
| N | Item | Yes | Can't Tell | No |
| 7 | Have ethical issues been taken into consideration? | X | | |
| 8 | Was the data analysis sufficiently rigorous? | X | | |
| 9 | Is there a clear statement of findings? | X | | |
| Section C: Will the results help locally? | | | | |
| N | Item | Yes | Can't Tell | No |
| 10 | How valuable is the research? | X | | |
| APPRAISAL SUMMARY | | | | |
| Positive/Methodologically sound | Negative/Relatively poor methodology | | | Unknowns |
| X | | | | |

Cohort Studies

| | | | | |
|---|---|---|---|---|
| Reference: Tusa et al., 2020 | | | | |
| Section A: Are the result valid? | | | | |
| N | Item | Yes | Can't Tell | No |
| 1 | Did the study address a clearly focused issue? | X | | |
| 2 | Was the cohort recruited in an acceptable way? | X | | |
| 3 | Was the exposure accurately measured to minimise bias? | X | | |
| 4 | Was the outcome accurately measured to minimise bias? | X | | |
| 5a | Have the authors identified all important confounding factors? | X | | |
| 5b | Have they taken account of the confounding factors in the design and/or analysis? | X | | |
| 6a | Was the follow up of subjects complete enough? | | | X |
| 6b | Was the follow up of subjects long enough? | | | X |
| Section B: What are the results? | | | | |
| N | Item | Yes | Can't Tell | No |
| 7 | What are the results of this study? | X | | |
| 8 | How precise are the results? | X | | |

| | | | | |
|---|---|---|---|---|
| 9 | Do you believe the results? | X | | |
| Section C: Will the results help locally? | | | | |
| N | Item | Yes | Can't Tell | No |
| 10 | Can the results be applied to the local population? | X | | |
| 11 | Do the results of this study fit with other available evidence? | X | | |
| 12 | What are the implications of this study for practice? | X | | |
| APPRAISAL SUMMARY | | | | |
| Positive/Methodologically sound | Negative/Relatively poor methodology | | | Unknowns |
| X | | | | |

| | | | | |
|---|---|---|---|---|
| Reference: Alshammari et al., 2020 | | | | |
| Section A: Are the result valid? | | | | |
| N | Item | Yes | Can't Tell | No |
| 1 | Did the study address a clearly focused issue? | X | | |
| 2 | Was the cohort recruited in an acceptable way? | | | X |
| 3 | Was the exposure accurately measured to minimise bias? | X | | |
| 4 | Was the outcome accurately measured to minimise bias? | X | | |
| 5a | Have the authors identified all important confounding factors? | X | | |
| 5b | Have they taken account of the confounding factors in the design and/or analysis? | X | | |
| 6a | Was the follow up of subjects complete enough? | | | X |
| 6b | Was the follow up of subjects long enough? | | | X |
| Section B: What are the results? | | | | |
| N | Item | Yes | Can't Tell | No |
| 7 | What are the results of this study? | X | | |
| 8 | How precise are the results? | X | | |
| 9 | Do you believe the results? | X | | |
| Section C: Will the results help locally? | | | | |
| N | Item | Yes | Can't Tell | No |
| 10 | Can the results be applied to the local population? | X | | |
| 11 | Do the results of this study fit with other available evidence? | X | | |
| 12 | What are the implications of this study for practice? | X | | |
| APPRAISAL SUMMARY | | | | |

| Positive/Methodologically sound | Negative/Relatively poor methodology | Unknowns |
|---|---|---|
| X | | |

| Reference: Jortberg et al., 2019 | | | | |
|---|---|---|---|---|
| Section A: Are the result valid? | | | | |
| N | Item | Yes | Can't Tell | No |
| 1 | Did the study address a clearly focused issue? | X | | |
| 2 | Was the cohort recruited in an acceptable way? | X | | |
| 3 | Was the exposure accurately measured to minimise bias? | X | | |
| 4 | Was the outcome accurately measured to minimise bias? | X | | |
| 5a | Have the authors identified all important confounding factors? | X | | |
| 5b | Have they taken account of the confounding factors in the design and/or analysis? | X | | |
| 6a | Was the follow up of subjects complete enough? | X | | |
| 6b | Was the follow up of subjects long enough? | X | | |
| Section B: What are the results? | | | | |
| N | Item | Yes | Can't Tell | No |
| 7 | What are the results of this study? | X | | |
| 8 | How precise are the results? | X | | |
| 9 | Do you believe the results? | X | | |
| Section C: Will the results help locally? | | | | |
| N | Item | Yes | Can't Tell | No |
| 10 | Can the results be applied to the local population? | X | | |
| 11 | Do the results of this study fit with other available evidence? | X | | |
| 12 | What are the implications of this study for practice? | X | | |
| APPRAISAL SUMMARY | | | | |
| Positive/Methodologically sound | Negative/Relatively poor methodology | | | Unknowns |
| X | | | | |

Case Control Study

| Reference: Oliveira et al., 2024 | | | | |
|---|---|---|---|---|
| Section A: Are the result valid? | | | | |
| N | Item | Yes | Can't Tell | No |
| 1 | Did the study address a clearly focused issue? | X | | |
| 2 | Did the authors use an appropriate method to answer their question? | X | | |
| 3 | Were the cases recruited in an acceptable way? | X | | |
| 4 | Were the controls selected in an acceptable way? | X | | |
| 5 | Was the exposure accurately measured to minimise bias? | X | | |
| 5b | Have they taken account of the confounding factors in the design and/or analysis? | X | | |
| 6a | Aside from the exposure, did the groups have similar characteristics? | X | | |
| 6b | Have the authors taken account of the potential confounding factors in the design and/or in their analysis? | X | | |
| Section B: What are the results? | | | | |
| N | Item | Yes | Can't Tell | No |
| 7 | Was the treatment effect large? | X | | |
| 8 | Was the estimate of the treatment effect precise? | X | | |
| 9 | Do you believe the results? | X | | |
| Section C: Will the results help locally? | | | | |
| N | Item | Yes | Can't Tell | No |
| 10 | Can the results be applied to your patients/the population of interest? | X | | |
| 11 | Do the results of this study fit with other available evidence? | X | | |
| APPRAISAL SUMMARY | | | | |
| Positive/Methodologically sound | Negative/Relatively poor methodology | | | Unknowns |
| X | | | | |

| Reference: Nagykaldi et al., 2020 | | | | |
|---|---|---|---|---|
| Section A: Are the result valid? | | | | |
| N | Item | Yes | Can't Tell | No |
| 1 | Did the study address a clearly focused issue? | X | | |
| 2 | Did the authors use an appropriate method to answer their question? | X | | |
| 3 | Were the cases recruited in an acceptable way? | X | | |

| | | | | |
|---|---|---|---|---|
| 4 | Were the controls selected in an acceptable way? | X | | |
| 5 | Was the exposure accurately measured to minimise bias? | X | | |
| 5b | Have they taken account of the confounding factors in the design and/or analysis? | X | | |
| 6a | Aside from the exposure, did the groups have similar characteristics? | X | | |
| 6b | Have the authors taken account of the potential confounding factors in the design and/or in their analysis? | X | | |
| Section B: What are the results? | | | | |
| N | Item | Yes | Can't Tell | No |
| 7 | Was the treatment effect large? | X | | |
| 8 | Was the estimate of the treatment effect precise? | X | | |
| 9 | Do you believe the results? | X | | |
| Section C: Will the results help locally? | | | | |
| N | Item | Yes | Can't Tell | No |
| 10 | Can the results be applied to your patients/the population of interest? | X | | |
| 11 | Do the results of this study fit with other available evidence? | X | | |
| APPRAISAL SUMMARY | | | | |
| Positive/Methodologically sound | Negative/Relatively poor methodology | | | Unknowns |
| X | | | | |

| | | | | |
|---|---|---|---|---|
| Reference: Dickinson et al., 2019 | | | | |
| Section A: Are the result valid? | | | | |
| N | Item | Yes | Can't Tell | No |
| 1 | Did the study address a clearly focused issue? | X | | |
| 2 | Did the authors use an appropriate method to answer their question? | X | | |
| 3 | Were the cases recruited in an acceptable way? | X | | |
| 4 | Were the controls selected in an acceptable way? | X | | |
| 5 | Was the exposure accurately measured to minimise bias? | X | | |
| 5b | Have they taken account of the confounding factors in the design and/or analysis? | X | | |
| 6a | Aside from the exposure, did the groups have similar characteristics? | X | | |
| 6b | Have the authors taken account of the potential confounding factors in the design and/or in their analysis? | X | | |
| Section B: What are the results? | | | | |

| N | Item | Yes | Can't Tell | No |
|---|---|---|---|---|
| 7 | Was the treatment effect large? | X | | |
| 8 | Was the estimate of the treatment effect precise? | X | | |
| 9 | Do you believe the results? | X | | |
| Section C: Will the results help locally? | | | | |
| N | Item | Yes | Can't Tell | No |
| 10 | Can the results be applied to your patients/the population of interest? | X | | |
| 11 | Do the results of this study fit with other available evidence? | X | | |
| APPRAISAL SUMMARY | | | | |
| Positive/Methodologically sound | Negative/Relatively poor methodology | | | Unknowns |
| X | | | | |

| Reference: Casanova et al., 2017 | | | | |
|---|---|---|---|---|
| Section A: Are the result valid? | | | | |
| N | Item | Yes | Can't Tell | No |
| 1 | Did the study address a clearly focused issue? | X | | |
| 2 | Did the authors use an appropriate method to answer their question? | X | | |
| 3 | Were the cases recruited in an acceptable way? | X | | |
| 4 | Were the controls selected in an acceptable way? | X | | |
| 5 | Was the exposure accurately measured to minimise bias? | X | | |
| 5b | Have they taken account of the confounding factors in the design and/or analysis? | X | | |
| 6a | Aside from the exposure, did the groups have similar characteristics? | X | | |
| 6b | Have the authors taken account of the potential confounding factors in the design and/or in their analysis? | X | | |
| Section B: What are the results? | | | | |
| N | Item | Yes | Can't Tell | No |
| 7 | Was the treatment effect large? | X | | |
| 8 | Was the estimate of the treatment effect precise? | X | | |
| 9 | Do you believe the results? | X | | |
| Section C: Will the results help locally? | | | | |
| N | Item | Yes | Can't Tell | No |
| 10 | Can the results be applied to your patients/the population of interest? | X | | |

| | | | | |
|---|---|---|---|---|
| 11 | Do the results of this study fit with other available evidence? | X | | |
| APPRAISAL SUMMARY | | | | |
| Positive/Methodologically sound | Negative/Relatively poor methodology | | | Unknowns |
| X | | | | |
